# Genome sequence of *Erwinia amylovora* bacteriophage Omen

**DOI:** 10.1128/mra.00122-24

**Published:** 2024-03-25

**Authors:** Gabriel Abreu, Edgar Garcia, Ana Oliveira, Hugo Oliveira

**Affiliations:** 1 Centre of Biological Engineering, University of Minho, Braga, Portugal; Portland State University, Portland, Oregon, USA

**Keywords:** *Erwinia*, bacteriophages

## Abstract

We report the genome of *Erwinia amylovora* phage Omen, isolated from a Portuguese orchard. Omen has a genome size of 85,304 bp, belongs to the genus *Kolesnikvirus* (myovirus morphotype), and shares over 80% nucleotide identity with various *Erwinia* phage genomes.

## ANNOUNCEMENT

*Erwinia amylovora* is responsible for devastating fire blight disease in various members of the *Rosaceae* family, such as apple and pear (1). Conventional methods used to control fire blight (chemical-based) are not effective and pose adverse effects on the environment (2, 3). Among the most promising alternative strategies are bacteriophages, which are viruses that specifically infect and kill their bacterial hosts (4, 5).

Omen was isolated from an orchard in Bombarral e Vale Covo, Portugal (surface soil sample collected on February 2023) using an enrichment protocol (6). Briefly, soil was diluted in SM buffer, mixed with an equal volume of 2× in lysogeny broth, spiked with *E. amylovora* CFBP 1232, and incubated overnight (28°C). After centrifugation, drops from the supernatant were spotted on bacterial lawns, using the same *Erwinia* strain used for enrichment. Phage plaques were purified several times from a single plaque amplified and filtered (0.2 µm). The phage morphology was assessed through a transmission electron microscope in a Jeol JEM 1400 (measurements of five viral particles were taken) with uranyl acetate staining as previously described (7). The genomic DNA was extracted from lysates (10^8^ PFU/mL) using the phenol/chloroform protocol (8) and quantified using NanoDrop2000 Spectrophotometer. DNA library (350 bp) was constructed with the VAHTS Universal Plus DNA Library Prep Kit for Illumina (ND617-02) according to the manufacturer’s protocol. The library quality was evaluated using Agilent Bioanalyzer 2100 and qPCR. The constructed library was sequenced on the Illumina Novaseq platform in PE150 mode. Base calling accuracy was determined by the Phred quality score (Q score). The sequence reads were then *de novo* assembled using the Geneious Prime v.2019.2.3 (Biomatters Ltd) using a medium-low sensitivity setting. Assembly quality was performed using Geneious Prime by resulting a circular contig (9). The genome was annotated with Pharokka v.1.3.2 (10), and subsequently manually curated using BLASTp against the non-redundant protein sequences database and HHpred against the Pfam-A_v36 database (E value of ≤10^−5^) (11, 12). The genome ends were determined with PhageTerm v.1.0.12 (13). Pairwise whole-genome nucleotide comparisons were made with BLASTn against the Nucleotide Collection Database (11). The lifestyle was predicted with PhageAI (https://app.phage.ai/, accessed on 1 February 2024). The presence of depolymerases was inferred with PhageDPO (bit.ly/phagedpo, accessed on 1 February 2024) (14). All tools were run with default parameters unless otherwise specified.

**Fig 1:**
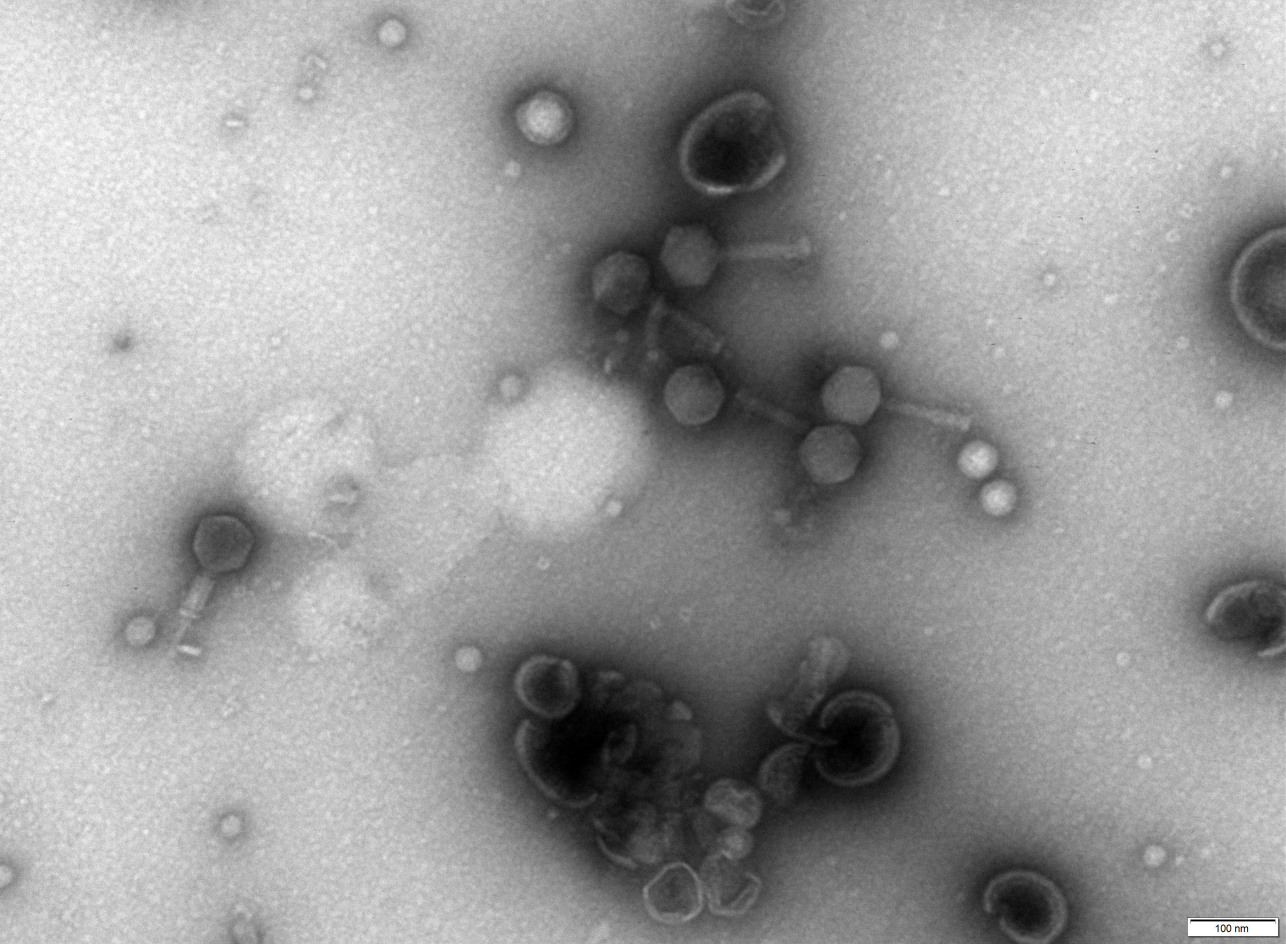
Transmission electron micrograph of *Erwinia amylovora*-infecting phage Omen, negatively stained with 2% uranyl acetate.

Omen has a myovirus-like morphotype (Fig. 1). Phage particles consist of a diameter isometric head (72 nm ± 5 nm vertex to vertex) and a nm-long contractile tail (112 ± 9 nm in length). The genome was assembled with 40,297 reads into a single 85.304 bp contig with a 70× coverage. Omen is predicted to have a circular permuted genome, 134 coding sequences (CDSs), and 25 tRNAs. The G + C content is 43.7%. It shares high nucleotide identity (>80%) to many other *Erwinia* phages, such as Panisse (GenBank accession number OQ818703), phiEa21-4 (NC_011811), and Pistou (OQ818706), which belong to the *Ounavirinae* (subfamily) and *Kolesnikvirus* (genus). At the protein level, only 52 out of 133 CDS could be assigned a function. No exopolysaccharide depolymerases were detected, which are frequent in *Erwinia* phages (15).

## Data Availability

The genome sequence for phage Omen and associated information are available in the GenBank and SRA, under the accession numbers PP278848 and SRX23537248, respectively, and are also associated with BioProject accession PRJNA1072067.
